# Emergence of carbapenem-resistant *Acinetobacter baumannii* clonal complex 2 in multiple hospitals in São Paulo state, Brazil

**DOI:** 10.1128/aac.01865-24

**Published:** 2025-07-23

**Authors:** Amanda Yaeko Yamada, Andreia Rodrigues de Souza, Geraldine Madalosso, Denise Brandão de Assis, Flavia Aparecida de Moraes França, Marlon Benedito Nascimento Santos, Karoline Rodrigues Campos, Claudio Tavares Sacchi, Monique Ribeiro Tiba-Casas, Eneas Carvalho, Carlos Henrique Camargo

**Affiliations:** 1 Bacteriology Division, Instituto Adolfo Lutz 89119, São Paulo, Brazil; 2 Faculdade de Medicina da Universidade de São Paulo 37884 , São Paulo, Brazil; 3 Centro de Vigilância Epidemiológica, São Paulo, Brazil; 4 Grupo de Vigilância Epidemiológica VIII, Mogi das Cruzes, Brazil; 5 Strategic Laboratory, Rapid Response Center, Instituto Adolfo Lutz 89119, São Paulo, Brazil; 6 Instituto Butantan 196591, São Paulo, Brazil; University of Pittsburgh School of Medicine, Pittsburgh, Pennsylvania, USA

**Keywords:** carbapenem resistance, hospital infection, illumina sequencing, clonal complex 2, COVID-19

## Abstract

Carbapenem-resistant *Acinetobacter baumannii* (CRAB) is a common pathogen prevalent in Brazilian hospitals. Worldwide, dissemination of CRAB is associated with the Clonal Complex 2 (CC2); in South America, however, CC1, 15, 25, and 79 are the most prevalent clones. In July 2020, our reference laboratory received the first CC2 isolates from a COVID-19 hospital, and, in the following months, this clone was detected in 15 other Brazilian institutions. To understand the clonal structure of this emerging pathogen, we characterize 89 isolates by whole-genome sequencing and antimicrobial susceptibility testing. Disk diffusion revealed resistance to all beta-lactams, aminoglycosides, fluoroquinolones, folate pathway antagonists, and tetracyclines, but susceptibility to polymyxin B. Resistome analysis identified diverse antimicrobial resistance genes, including the *bla*_OXA-23_ associated with Tn*2006*, and *armA* in AbGRI3, conferring resistance to beta-lactams and aminoglycosides, respectively. Fine-scale phylogeny based on single nucleotide polymorphisms (SNPs) revealed that Brazilian CRAB CC2 isolates were closely related, presenting up to 755 SNPs in pairwise comparison. We did not observe hospital-specific subclones, indicating multiple introductions and/or inter-hospital dissemination. This study reports the rapid arrival and spread of CRAB CC2 isolates in multiple hospitals, likely driven by infection control deficiencies experienced during the COVID-19 pandemic.

## INTRODUCTION

Carbapenem-resistant *Acinetobacter baumannii* (CRAB) is a critical pathogen associated with high mortality rates, particularly among at-risk patients (1). Designated as a priority pathogen by the World Health Organization, the urgent development of novel antibiotics is imperative to combat CRAB (2). This microorganism has become a leading cause of healthcare-associated infections (HAI), especially in intensive care units, where it is recognized for severe outbreaks linked to carbapenem-resistant clonal dissemination (3).

According to the SENTRY Antimicrobial Surveillance Program in 1997, a marked decline in *A. baumannii* susceptibility to carbapenems (imipenem and meropenem) has been documented. During 2009–2016, susceptibility rates reached their lowest levels in Latin America (13.7%–14.4%), Europe (22.2%–23.7%), Asia-Pacific (18.4%–18.5%), and North America (43.7%–46.8%) (4). The COVID-19 pandemic further exacerbated the global prevalence of CRAB, with significant outbreaks reported worldwide. In Italy, infections increased by 5.5-fold (5), while in the United States, hospital-onset CRAB rates surged by 78.0% in 2020 (6). In Brazil, a major hospital in São Paulo reported a 108% increase in HAI caused by CRAB between 2017–2019 and 2020 (7).

CRAB is known for its strong clonal dissemination, primarily driven by specific global lineages. Among them, the Clonal Complex (CC) CC2 is the most prevalent worldwide, followed by CC79, which is considered an endemic lineage in Latin America (also known as the “Pan-American clone”) (3, 8). During the first wave of the COVID-19 pandemic, our reference laboratory in São Paulo, Brazil, identified the first isolates of CRAB belonging to CC2 as part of a large outbreak (9). Subsequently, additional CC2 isolates were detected in 15 other hospitals across the region.

Given the critical emergence of CRAB CC2 in Brazil, this study aims to evaluate the antimicrobial susceptibility profiles and clonal relationships of CRAB CC2 isolates recovered from patients in various hospitals during the pre-vaccination phase of the COVID-19 pandemic (2020–2021).

## MATERIALS AND METHODS

### Isolates

Between January 2020 and December 2021, 1,867 Gram-negative bacterial isolates were sent to our reference laboratory for species identification (by MALDI-TOF MS, Bruker Daltonics, Bremen, Germany) and antimicrobial susceptibility testing. Seven hundred and ninety-one (791) were identified as *A. baumannii*. Additionally, to initial tests, *A. baumannii* isolates had their clonal complexes determined by a multiplex PCR (10).

### Identification of CRAB clonal complexes

CRAB clonal complexes were identified using a multiplex PCR (m3LST) protocol targeting three specific genes: *ompA* (outer membrane porin A), *csuE* (biofilm formation), and *bla*_OXA-51-like_ (encoding carbapenemase) (10). The *bla*_OXA-51-like_ gene was fully sequenced to determine variants associated with CRAB clonal complexes (11).

### Pulsed-Field gel electrophoresis

Isolates were genotyped using ApaI-Pulsed-Field Gel Electrophoresis (PFGE) on a CHEF-DR III system (Bio-Rad, Hercules, USA). The run time was 19 hours with switch times of 5–20 seconds at 6 V/cm (12). The Lambda PFG ladder marker (New England Biolabs, Ipswich, USA) was used for normalization. Gel images were analyzed with BioNumerics 7.6.2 software (Applied Maths, Sint-Martens-Latem, Belgium) to generate a dendrogram using the Unweighted Pair Group Method with Arithmetic average clustering method, with a tolerance and optimization of 1.5%.

### Antimicrobial susceptibility testing (AST)

Antimicrobial susceptibility was assessed using the disk-diffusion test (13), following the Brazilian version of the EUCAST guidelines (14). Antibiotics tested included amikacin (30 µg), ciprofloxacin (5 µg), gentamicin (10 µg), imipenem (10 µg), meropenem (10 µg), levofloxacin (5 µg), trimethoprim-sulfamethoxazole (1.25/23.75 µg), and tobramycin (10 µg). Minocycline (30 µg) was interpreted according to CLSI breakpoints (15). Minimum Inhibitory Concentrations (MICs) for tigecycline and ampicillin-sulbactam (2:1) were determined using epsilometer strips (Liofilchem, Italy) on Mueller-Hinton agar, following BrCAST guidelines. For tigecycline, the following breakpoints for *Escherichia coli* were applied: ≤0.5 mg/L (susceptible) and >0.5 mg/L (resistant). Polymyxin B MICs were determined via broth microdilution, with resistance defined as MIC ≥4 mg/L (14).

### Identification of resistance genes

Multiplex PCR was performed to detect resistance genes, including *bla*_OXA-23-like_, *bla*_OXA-24-like_, *bla*_OXA-51-like_, *bla*_OXA-58-like_, and *bla*_OXA-143-like_ (16, 17), as well as *bla*_KPC_, *bla*_NDM_, and *bla*_OXA-48_ (18).

### DNA extraction

Genomic DNA was extracted using the PureLink Genomic DNA kit (Invitrogen, Waltham, USA) for short reads and using the Wizard kit (Promega, Madison, EUA) for long reads, both from 1.4 mL of an overnight (18 h) bacterial cultures growth on Luria Bertani broth (Difco, United Kingdom) at 35°C. DNA concentration and purity were assessed using a NanoDrop One spectrophotometer (Thermo Fisher Scientific, Waltham, USA) and a Qubit fluorometer (Thermo Fisher Scientific, Waltham, USA). DNA integrity was evaluated using electrophoresis on ethidium bromide-stained 2% agarose gels.

### Library preparation and whole-genome sequencing (short reads)

Genomic libraries were prepared using DNA Prep (Illumina, San Diego, USA) with magnetic beads to cleave and tag DNA. PCR amplification was performed with the Nextera DNA CD Indexes kit (Illumina, San Diego, USA). Sequencing was conducted using an Illumina MiSeq platform (Illumina, San Diego, USA) at the Strategic Laboratory, Adolfo Lutz Institute, generating 75 bp paired-end reads.

### Library preparation and whole-genome sequencing (long reads)

DNA shearing was performed using g-TUBEs (Covaris, Woburn, Massachusetts) to obtain fragments averaging 15–20 kb. Short fragments were removed by size selection using SMRTbell Beads (Pacific Biosciences, California, EUA), followed by quality control with Qubit and fragment length assessment via capillary electrophoresis on a QIAxcel system (Qiagen, Hilden, Germany). Library preparation was performed using the SMRTbell Express Template Prep Kit 2.0 (Pacific Biosciences), including end-repair, ligation of SMRTbell adapters, exonuclease treatment to remove incomplete or unligated products, and final purification and size selection using AMPure PB beads to retain fragments >10–15 kb. The library was quantified using Qubit and validated with QIAxcel. Sequencing was performed on the PacBio Sequel IIe system using a SMRT Cell 8M, with the Circular Consensus Sequencing mode to generate HiFi reads (~Q40) ranging from 8–20 kb in length.

### Quality control (QC) and genome assembly

Reads were evaluated for quality and GC content using FastQC (version 0.12.1). Contamination screening was performed with Kraken2 (version 1.1.1) (19) via the Galaxy Europe Server. Genome assembly was conducted using CLC Workbench (Qiagen, Venlo, Netherlands), and QC metrics (e.g., genome size, N50, N90, contig count, and GC content) were determined using QUAST (version 5.2.0) (20, 21). CheckM (version 1.2.3, https://github.com/chklovski/CheckM2) was employed to assess completeness and contamination in the sequenced genomes (22) (Table S1). Hybrid genome was assembled using Unicycler (https://github.com/rrwick/Unicycler).

### Genome analysis

Species identification was confirmed via Average Nucleotide Identity with a cutoff of >95% (23). Sequence types (STs) were assigned using PubMLST for Pasteur and Oxford schemes (https://pubmlst.org/organisms/acinetobacter-baumannii). Resistance genes and mutations were identified using ResFinder (version 4.1—https://cge.food.dtu.dk/services/ResFinder/) and CARD (version 6.0.2—https://card.mcmaster.ca/analyze/rgi). Capsular polysaccharide (KL) and outer core locus (OCL) gene clusters were analyzed using Kaptive (version 2.0.4—https://kaptive-web.erc.monash.edu/jobs). Resistance islands AbGRI1 to AbGRI3 were investigated to define the context of resistance genes (*armA*, *strA*, *strB*, *tetB*) by automatically annotating the genome with Prokka, followed by manual curation in BioNumerics v.7.6 after BLAST analysis of each ORF. The genetic location of *bla*_OXA-23_ was also assessed using a representative genome (1238_21), which was sequenced with both short and long reads. The IslandViewer 4 web server was used for the prediction and interactive visualization of genomic islands (regions of probable horizontal origin) (https://www.pathogenomics.sfu.ca/islandviewer/).

### Mapping to the reference genome

Long-read sequencing of one isolate enabled the mapping of regions not detected in some isolates due to the limitations of short-read sequencing. For this purpose, mapping was performed against the reference genome (1238_21), focusing on the regions containing the *bla*_OXA-23_ gene and the AbGRI1-1 genomic island. Mapping was conducted using Bowtie2 with the "very-sensitive-local" option, and coverage was determined with Samtools for the two copies of *bla*_OXA-23_ in all isolates. For the identification of AbGRI1-1, the BWA software was used.

### Phylogenetic analyses

Local phylogenetic analysis was performed on genomes generated from this study (Table S1). Genomes were annotated using Prokka (version 1.14.6) (24, 25), and pan-genome analysis was performed with Roary (version 3.13.0) (26). Core genome SNPs were extracted using SNP-sites (version 2.5.1) (27), and phylogenetic inference was performed using RAxML (version 1.0.0—https://cme.h-its.org/exelixis/web/software/raxml/) with 1,000 bootstrap replicates and the GRT + Gamma substitution model. Visualization was achieved using Microreact (28). SNP-distance matrices were computed using SNP-dists (version 0.8.2—https://github.com/tseemann/snp-dists).

A second analysis, following the same methodology described above, included ST218 isolates with capsular type KL7 available in PubMLST and PathogenWatch (duplicates removed and up to two genomes per country and per year were selected; *n* = 27), as well as genomes reported by Morgado et al. (29) (*n* = 5) and Camargo et al. (9) (*n* = 1), along with the isolates from the present study (*n* = 89), resulting in a phylogeny comprising 122 genomes (9, 29).

## RESULTS

### CRAB clonal complexes

During the study period, 661 non-duplicate *A. baumannii* isolates recovered from 10 municipalities within the state of São Paulo, Brazil, were analyzed. Among these, 63.9% (422/661) were from male patients, and one isolate was from a newborn with unspecified sex. The 661 isolates were distributed into clonal complexes as follows: CC1 (22.2%; 147/661), CC2 (33.3%; 220/661), CC15 (7.0%; 46/661), CC25 (3.8%; 25/661), CC79 (27.8%; 184/661), and others/unidentified (5.9%; 39/661). All CC2 isolates (220/661), which also carried the intrinsic *bla*_OXA-66_ allele, were collected from 16 different hospitals across five cities in São Paulo state. They were predominantly recovered from tracheal secretions (37.3%) and blood (24.5%), with additional sources including surveillance swabs (21.4%), urine (12.3%), wound secretions (0.9%), tissue fragments (0.9%), and pleural lavage (0.45%). Due to the limited discriminatory power of PFGE (which showed >87% overall similarity; data not shown), up to 10 isolates per hospital were randomly selected for subsequent analyses, resulting in a total of 89 isolates from 16 hospitals.

### Antimicrobial susceptibility

All 89 CC2 isolates randomly selected for further analysis were resistant to carbapenems (imipenem and meropenem), fluoroquinolones (ciprofloxacin and levofloxacin), β-lactam/β-lactamase inhibitor combinations (ampicillin-sulbactam), and the glycylcycline tigecycline. None of the isolates exhibited resistance to polymyxin B, with MIC50 and MIC90 values at 0.5 and 1.0 mg/L (Table 1). Minocycline was the second most effective antimicrobial, with 30.3% of isolates classified as non-resistant.

**TABLE 1:** Antimicrobial susceptibility of clinical isolates of carbapenem-resistant *A. baumannii* CC2 from São Paulo state, recovered between 2020 and 2021 (*n* = 89)^*a*^*^,b^*

Antimicrobial agent	Method	Breakpoint	%S	%I	%R	MIC50	MIC90	MIC range
Amikacin	Disk diffusion	BrCAST	4.5	0.0	95.5	–	–	–
Gentamicin	Disk diffusion	BrCAST	4.5	0.0	95.5	–	–	–
Tobramycin	Disk diffusion	BrCAST	2.2	0.0	97.8	–	–	–
Imipenem	Disk diffusion	BrCAST	0.0	0.0	100.0	–	–	–
Meropenem	Disk diffusion	BrCAST	0.0	0.0	100.0	–	–	–
Ciprofloxacin	Disk diffusion	BrCAST	0.0	0.0	100.0	–	–	–
Levofloxacin	Disk diffusion	BrCAST	0.0	0.0	100.0	–	–	–
Trimethoprim-sulfamethoxazole	Disk diffusion	BrCAST	2.2	0.0	97.8	–	–	–
Minocycline	Disk diffusion	CLSI	1.1	29.2	69.7	–	–	–
Polymyxin B	Broth microdilution	BrCAST	100.0	0.0	0.0	0.5	1.0	0.25 to 2.0
Tigecycline	Gradient strip	BrCAST	0.0	0.0	100.0	3.0	3.0	2.0 to 4.0
Ampicillin-sulbactam	Gradient strip	BrCAST	0.0	0.0	100.0	>256	>256	32.0 to >256

^*a*^ The minimum inhibitory concentration (MIC) values that inhibit the growth of 50% (MIC50) and 90% (MIC90) of the isolates are indicated, as well as the MIC range, in mg/L. S susceptible, I intermediate, R resistant.

^*b*^ “–”, Not applicable.

### Resistance gene profile

PCR analysis detected the carbapenemase gene *bla*_OXA-23-like_ and the intrinsic *bla*_OXA-51-like_ in all isolates, while *bla*_OXA-24-like_, *bla*_OXA-48-like_, *bla*_OXA-58-like_, *bla*_OXA-143-like_, *bla*_KPC_, and *bla*_NDM_ were not detected.

### Sequencing and genomic characterization

**Fig 1:**
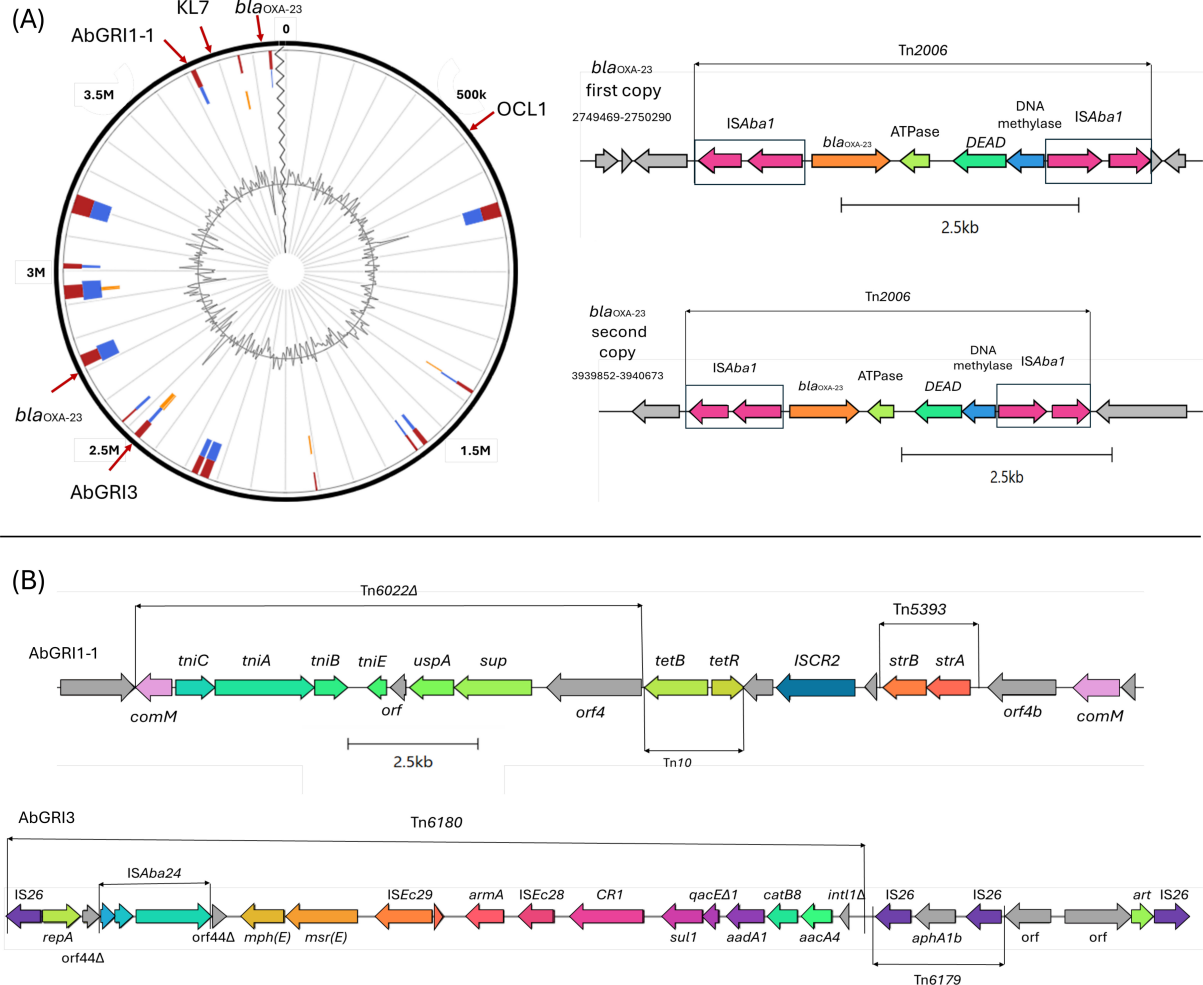
*A. baumannii* genome isolate 1238_21 (hybrid assembly) generated using IslandViewer4. (**A**) Genetic context associated with the two copies of *bla*_OXA-23_ gene, identified as part of Tn*2006*. Pink arrows indicate the inversely oriented IS*Aba1* elements flanking *bla*_OXA-23_. (**B**) Genetic context associated with AbGRI1-1 and AbGRI3. The structure of the AbGRI1-1 genomic island is shown, indicating the presence of *strA, strB, and tetB,* flanked by *comM* and parts of the transposons Tn*6022*∆, Tn*10*, and Tn*5393*. The AbGRI3 island harbors the *armA* gene within Tn*6180*, adjacent to Tn*6179*, with two IS*26* elements flanking the *aphA1b* gene. Island blocks indicate the prediction methods: Integrated (red), IslandPath-DIMOB (blue), and SIGI-HMM (orange).

Except for one isolate (1310_21) identified as ST2 (Pasteur)/ST3308 (Oxford), which has a single nucleotide alteration in a single allele of the *cpn60* gene in the Oxford scheme, all CRAB CC2 isolates were classified as ST2 (Pasteur)/ST218 (Oxford). All isolates harbored the capsular polysaccharide *KL7* locus and the outer core oligosaccharide *OCL1* locus. Among the sequenced isolates, almost all carried resistance-associated genes for aminoglycosides (*armA and aphA1*), sulfonamides (*sul1*), β-lactams (*bla*_OXA-23_), fluoroquinolones (mutations in *parC* and *gyrA*), and tetracycline (*tetB*) (Table 2). The genes *aphA6, floR, aadB*, and *sul2* were detected in only one isolate (Table 2). All isolates exhibited mutations *gyrA* S81L and *parC* S84L, V104I, and D105E, which confer fluoroquinolone resistance. The *armA* gene was located within AbGRI3 in all isolates carrying this determinant (*n* = 85/89), in contigs approximately 17.4 kb in size. AbGRI3 also harbored the *mph(E*), *msr(E*), *sul1*, *aadA1*, *catB8*, and *aacA4* genes (Fig. 1). The AbGRI1-1 resistance island was identified in 82/89 isolates, presenting a contig of at least 8 kb containing the *strA*, *strB*, and *tetB* resistance genes. In 7/89 isolates where this region was not detected in the assembled genome, read mapping was performed against the hybrid assembly of isolate 1238_21. Consequently, the AbGRI1-1 region was identified in all 89 isolates (Fig. 1). Regarding the genetic context of the carbapenemase gene *bla*_OXA-23_, Tn*2006* was identified in the hybrid assembly as two copies located at positions 2,750,323–2,751,144 bp and 3,940,705–3,941,521 bp on the chromosome (Fig. 1). Tn*2006* is composed of two inversely oriented IS*Aba1* elements flanking an internal segment containing *bla*_OXA-23_ (Fig. 1). These regions, as identified in the hybrid genome 1238_21, were mapped against the reads of other genomes where only one copy had initially been identified. The presence of two copies was confirmed in all 89 genomes, along with the flanking IS*Aba1* elements, characterizing all as Tn*2006*.

**TABLE 2:** Frequency of resistance genes in clinical isolates of carbapenem-resistant A. baumannii CC2 from São Paulo state, recovered between 2020 and 2021 (*n* = 89)

Antimicrobial categories	Resistance genes	No. of isolates (%)
Aminoglycosides	*aadB*	1 (1.1%)
	*aphA1*	83 (93.2%)
	*aphA6*	2 (2.2%)
	*armA*	85 (95.5%)
β-lactams	*bla*_OXA-23_	89 (100%)
Fluoroquinolones	*gyrA* (S81L)	89 (100%)
	*parC* (S84L, V104I, and D105E)	89 (100%)
Sulfonamides	*sul1*	85 (95.5%)
	*sul2*	1 (1.1%)
Tetracycline	*tetB*	89 (100%)

### Phylogenetic analysis

#### Local phylogeny

**Fig 2:**
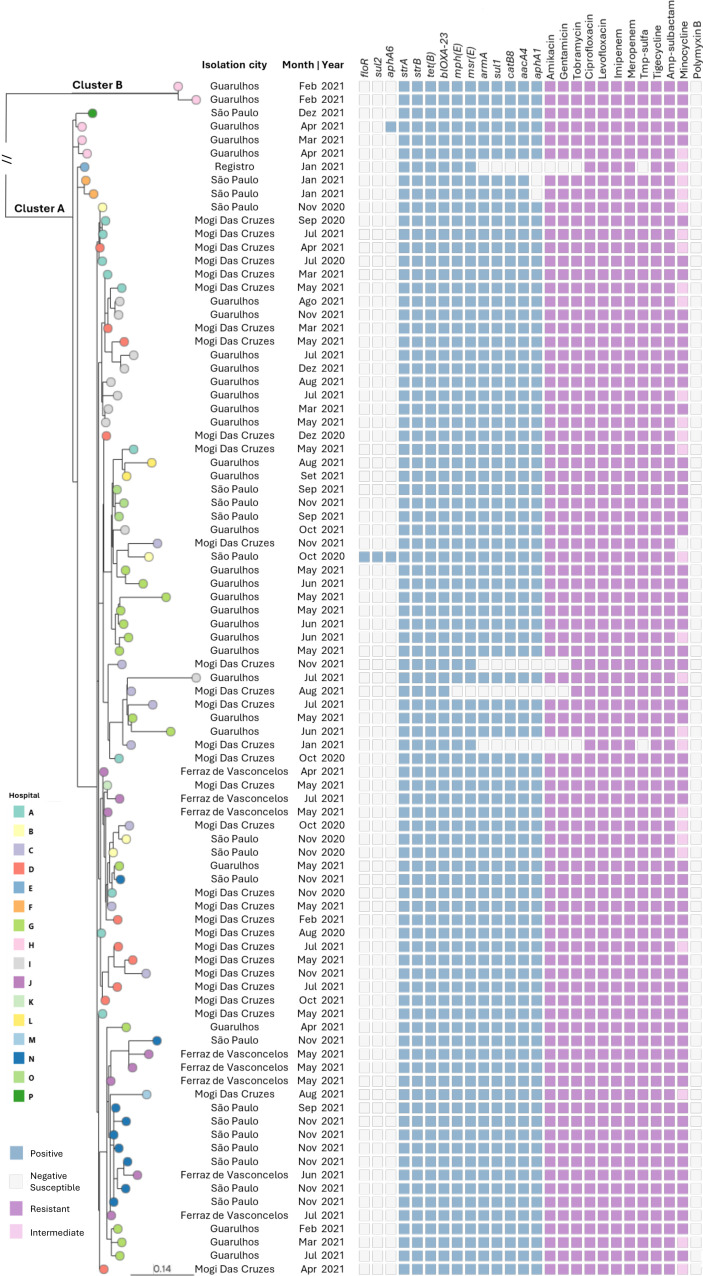
Phylogenetic relationship of 89 CRAB CC2 isolates from this study. Image generated by Microreact and metadata is available from Table S1.

The first phylogenetic analysis of the 89 genomes generated in this study identified a highly similar subclade (Cluster A), comprising 87 isolates with a minimum of zero (between 1270/21 and 1271/21) and a maximum of 104 SNPs between them. These genomes clustered independently of the hospital, city, isolation source, or time of isolation. A second subclade (Cluster B), consisting of two isolates (229_21 and 230_21) from the same hospital, was identified, sharing 19 SNPs (Fig. 2). Pairwise comparisons among all 89 genomes revealed a total of 755 SNPs (Table S3).

#### Global phylogeny

A second phylogenetic analysis, including 32 global ST218 KL7 genomes along with the isolates from this study, revealed a high genetic diversity, between zero (among the same 1270_21 and 1271_21 sequenced in this study) and up to 2,380 identified SNPs. Three strains from China were responsible for this high SNP value (SAMN14943625, SAMN14943607, and SAMN15538698). When the analysis is carried out without those isolates, the variation is between zero and 985 SNPs (Table S4). Brazilian isolates from the states of Rio de Janeiro and São Paulo clustered closely together (ranging from zero to 125 SNPs), while two São Paulo isolates (229_21 and 230_21) clustered with international isolates from Portugal, Canada, Taiwan, Singapore, Hungary, or Italy (Fig. S1).

## DISCUSSION

This study reports the emergence of the CRAB CC2 lineage in multiple hospitals across São Paulo, Brazil, during the early COVID-19 pandemic. In addition to carbapenem resistance, this clone exhibited high-level aminoglycoside resistance mediated by the *armA* gene, previously reported only in sporadic *A. baumannii* isolates from Brazil.

CRAB is a significant pathogen in healthcare settings, frequently implicated in hospital outbreaks (30). According to Br-GLASS surveillance data, *A. baumannii* isolates in Brazil exhibit high antimicrobial resistance rates, including 81.4% resistance to meropenem, 86.8% to ciprofloxacin, and 63% to amikacin (31). Consistent with these findings, most of the isolates were resistant to all evaluated antimicrobial agents (except polymyxin B). Despite its efficacy, polymyxin B monotherapy is not recommended due to limited clinical effectiveness and associated mortality risks (32–34).

The resistance genes detected largely explain the resistance profiles observed *in vitro*. The predominance of two copies of the *bla*_OXA-23_ carbapenemase gene and the *armA* 16S rRNA methylase gene in these isolates highlights their extensive resistance mechanisms (35, 36). Additional genes conferring resistance to tetracyclines, sulfonamides (*sul1*), and quinolones, along with mutations in *gyrA* and *parC*, were also identified, supporting the phenotypic findings. Most resistance genes were located within resistance islands, such as *armA* in AbGRI3 - largely disseminated in CC2 (37)—and *strA/strB* and *tetB* in AbGRI1-1 (38). The presence of resistance islands in the bacterial chromosome helps explain the multidrug-resistant phenotype observed in *Acinetobacter* over recent decades (4).

Before the COVID-19 pandemic, South America displayed distinct molecular epidemiology for CRAB, with clonal complexes CC1, CC15, CC25, and CC79 predominating, while CC2 was sporadically observed (39, 40). The clone CC2 was previously reported in sporadic cases across different states in Brazil, including isolates from 1999 to 2003 in Paraná (41), 2006 to 2007 in Rio de Janeiro (42), and 2013 to 2014 in Rio Grande do Sul states (43). After these descriptions, no additional cases of CC2 were reported in these regions until recently. Following the COVID-19 pandemic, however, CC2 was associated with multiple local outbreaks in São Paulo (9), where the mortality rate reached 68.6%, and in Rio de Janeiro (29, 44). Additionally, a nosocomial outbreak caused by carbapenem-resistant CRAB carrying *bla*_NDM-1_ was reported in a hospital in Paraná state, Southern Brazil (45, 46). This outbreak resulted in a high mortality rate (62%) and underscores the ability of this clone to accumulate multiple resistance mechanisms.

Our phylogenetic analysis revealed minimal SNP differences among isolates from distinct hospitals, suggesting widespread dissemination rather than hospital-specific outbreaks, likely driven by pandemic-related factors such as workforce shortages, overcrowding, and patient transfers (47). In the same way, we were unable to identify a resistance pattern associated with any hospital or city.

With the SNP analysis, we could observe that Brazilian isolates are clustered together, with two circulating lineages, and that they differ from isolates of other countries. Therefore, the source of this clone in Brazil remains unclear.

All isolates harbored the *KL7* capsular locus, which is linked to immune evasion and virulence. This locus has been identified in CC2 isolates from São Paulo and Rio de Janeiro, as well as in isolates from at least 12 other countries, suggesting a global phylogenetic connection (29, 44, 48). The *KL7* locus is associated with the biosynthesis of legionaminic acid, which enhances immune evasion and may contribute to the success of CC2 in clinical settings (48).

Although this study provides novel insights into the genomic and phenotypic characteristics of CRAB CC2, some limitations must be acknowledged. Isolate submission was voluntary, potentially underestimating its prevalence. Resource constraints limited the sample size, and the absence of clinical data hindered analyses of patient outcomes and transmission dynamics. In addition, only one isolate was submitted to long-read sequencing, due to financial restrictions.

This study demonstrates the emergence of the CRAB CC2 clone in São Paulo hospitals during the COVID-19 pandemic, highlighting its extensive resistance mechanisms and potential for dissemination. These findings reinforce the urgent need for enhanced infection control measures, robust genomic surveillance, and antimicrobial stewardship to mitigate the impact of highly resistant *A. baumannii* clones in Brazil, a country with high and endemic antimicrobial resistance rates (49, 50).

### Conclusions

We report the introduction and multi-hospital spread of a new *Acinetobacter baumannii* lineage, characterized as CC2:ST218:KL7 and producing OXA-23 and ArmA resistance determinants. The emergence of this novel lineage, with increased antimicrobial resistance and virulence factors, highlights the potential for clonal replacement of CRAB in São Paulo, carrying significant implications for therapeutic options and patient outcomes.

## Data Availability

The Whole Genome Shotgun project has been deposited at DDBJ/ENA/GenBank under the accession JBEQCV000000000-JBEQGF000000000 (Table S1).

## SUPPLEMENTAL MATERIAL

Fig. 1 aac.01865-24-s0001.pdf Phylogenetic relationship among global isolates of CRAB CC2.

Table S1 aac.01865-24-s0002.xlsx Metadata of the 89 isolates in this study.

Table S2 aac.01865-24-s0003.xlsx Data of the 122 global ST218/KL7 isolates.

Table S3 aac.01865-24-s0004.xlsx SNP distance matrix between the 89 isolates CRAB from this study, organized according to the phylogenetic tree.

Table S4 aac.01865-24-s0005.xlsx SNP distance matrix between the 122 global ST218/KL7 isolates, organized according to the phylogenetic tree.

ASM does not own the copyrights to Supplemental Material that may be linked to, or accessed through, an article. The authors have granted ASM a non-exclusive, world-wide license to publish the Supplemental Material files. Please contact the corresponding author directly for reuse.
