# Supplementary material for: Emergence of carbapenem-resistant Acinetobacter baumannii clonal complex 2 in multiple hospitals in São Paulo state, Brazil
Source: Antimicrob Agents Chemother. 2025 Jul 23;69(9):e01865-24. doi: 10.1128/aac.01865-24 (PMC12406670; doi:10.1128/aac.01865-24)
Supplement: Fig. 1 — Phylogenetic relationship among global isolates of CRAB CC2. [file aac.01865-24-s0001.pdf]

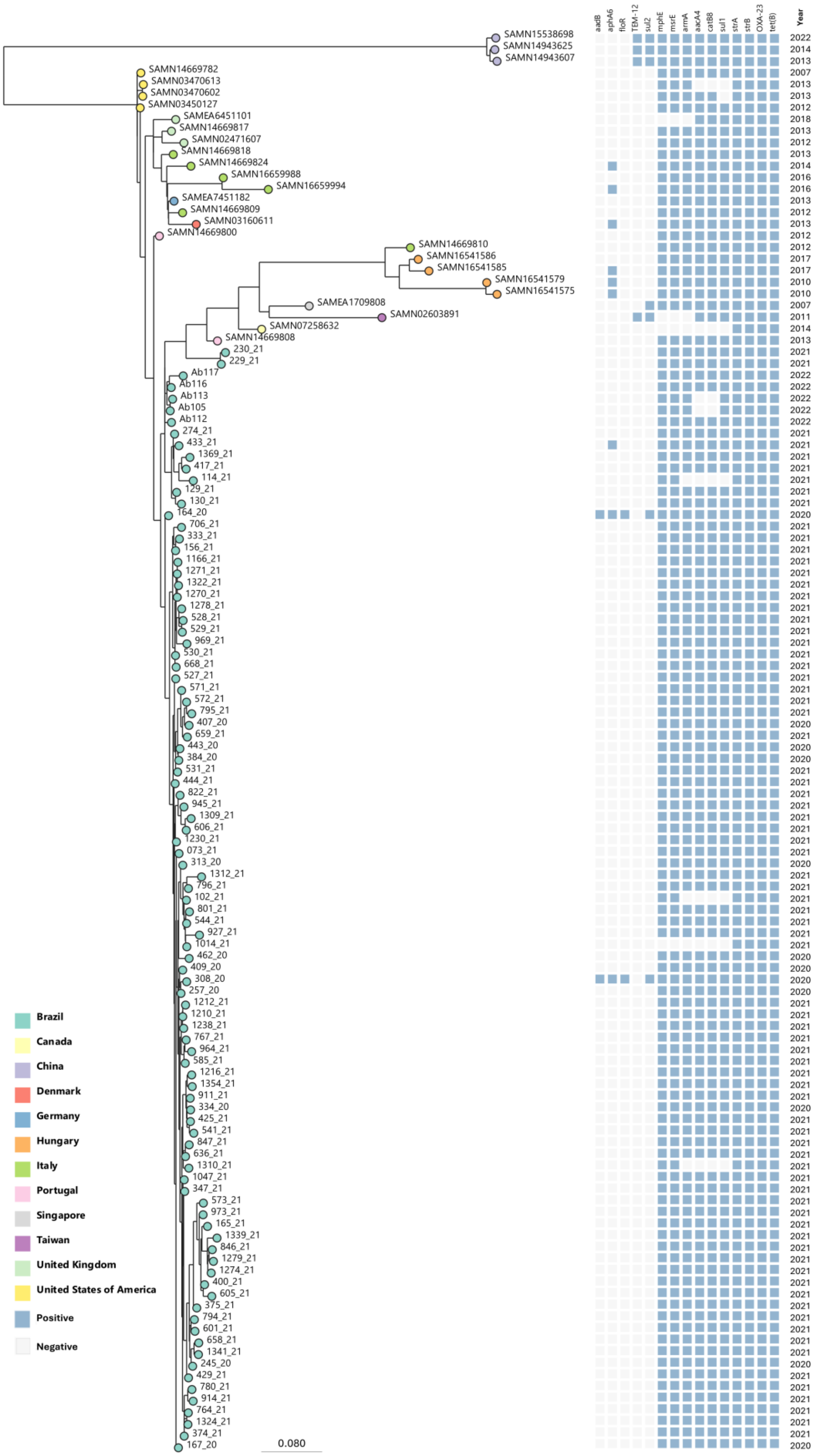

Supplementary Figure 1. Phylogenetic relationship among global isolates of CRAB CC2. Image generated by Microreact and metadata is available from Supplementary Table 2.
